# Supplementary material for: Cloning, expression and characterisation of antigen-specific recombinant bat immunoglobulin from the black flying fox (Pteropus alecto)
Source: Front Immunol. 2026 Mar 4;17:1743193. doi: 10.3389/fimmu.2026.1743193 (PMC12995615; doi:10.3389/fimmu.2026.1743193)
Supplement: Supplementary file 8 [file Table1.docx]

**Supplementary Table1.** Nucleotide sequences and plasmid maps of pNBF Bat 5B3 Light chain and pNBF Bat 5B3 heavy chain IgG1 with Bsp or Hsp.

| pNBF Bat 5B3 Light chain | GACGTTTAAACAATTCCTTAGGCTACCGGGTAGGGGAGGCGCTTTTCCCAAGGCAGTCTGGAGCATGCGCTTTAGCAGCCCCGCTGGGCACTTGGCGCTACACAAGTGGCCTCTGGCCTCGCACACATTCCACATCCACCGGCCGGTAGGCGCCAACCGGCTCCGTTCTTTGGTGGCCCCTTCGCGCCACCTTCTACTCCTCCCCTAGTCAGGAAGTTCCCCCCCGCCCCGCAGCTCGCGTCGTGCAGGACGTGACAAATGGAAGTAGCACGTCTCACTAGTCTCGTGCAGATGGACAGCACCGCTGAGCAATGGAAGCGGGTAGGCCTTTGGGGCAGCGGCCAATAGCAGCTTTGCTCCTTCGCTTTCTGGGCTCAGAGGCTGGGAAGGGGTGGGTCCGGGGGCGGGCTCAGGGGCGGGCTCAGGGGCGGGGCGGGCGCCCGAAGGTCCTCCGGAGGCCCGGCATTCTGCACGCTTCAAAAGCGCACGTCTGCCGCGCTGTTCTCCTCTTCCTCATCTCCGGGCCTTTCGACCAGCTTACC[Puromycin] ATGACCGAGTACAAGCCCACGGTGCGCCTCGCCACCCGCGACGACGTCCCCAGGGCCGTACGCACCCTCGCCGCCGCGTTCGCCGACTACCCCGCCACGCGCCACACCGTCGATCCGGACCGCCACATCGAGCGGGTCACCGAGCTGCAAGAACTCTTCCTCACGCGCGTCGGGCTCGACATCGGCAAGGTGTGGGTCGCGGACGACGGCGCCGCGGTGGCGGTCTGGACCACGCCGGAGAGCGTCGAAGCGGGGGCGGTGTTCGCCGAGATCGGCCCGCGCATGGCCGAGTTGAGCGGTTCCCGGCTGGCCGCGCAGCAACAGATGGAAGGCCTCCTGGCGCCGCACCGGCCCAAGGAGCCCGCGTGGTTCCTGGCCACCGTCGGCGTCTCGCCCGACCACCAGGGCAAGGGTCTGGGCAGCGCCGTCGTGCTCCCCGGAGTGGAGGCGGCCGAGCGCGCCGGGGTGCCCGCCTTCCTGGAGACCTCCGCGCCCCGCAACCTCCCCTTCTACGAGCGGCTCGGCTTCACCGTCACCGCCGACGTCGAGGTGCCCGAAGGACCGCGCACCTGGTGCATGACCCGCAAGCCCGGTGCC  TGACGCCCGCCCCACGACCCGCAGCGCCCGACCGAAAGGAGCGCACGACCCCATGCATCGTAGAATTCCGGACTTGACTCATGCTTGTTTCACTTTCACATGGAATTTCCCAGTTATGAAATTAATAAAAATCAATGGTTTCCACATCTCTCAGTGCCTCTATCTGGAGGCCAGGTAGGGCTGGCCTTGGGGGAGGGGGAGGCCAGAATGACTCCAAGAGCTACAGGAAGGCAGGTCAGAGACCCCACTGGACAAACAGTGGCTGGACTCTGCACCATAACACACAATCAACAGGGGAGTGAGCTGGAGGGGGATCGTTTAAACCTTAAGATATCCAG[SV40 Promoter] CATCTCAATTAGTCAGCAACCATAGTCCCGCCCCTAAC TCCGCCCATCCCGCCCCTAACTCCGCCCAGTTCCGCCCATTCTCCGCCCCATGGCTGACTAATTTTTTTTATTTATGCAGAGGCCGAGGCCGCCTCTGCCTCTGAGCTAGTCGCGATTGATCAGCGCCACCTTGGGGCCACCGCTCAGAGCACCTTCCACC [GS gene]ATGGCCACCTCAGCAAGTTCCCACTTGAACAAAAACATCAAGCAAATGTACTTGTGCCTGCCCCAGGGTGAGAAAGTCCAAGCCATGTATATCTGGGTTGATGGTACTGGAGAAGGACTGCGCTGCAAAACCCGCACCCTGGACTGTGAGCCCAAGTGTGTAGAAGAGTTACCTGAGTGGAATTTTGATGGCTCTAGTACCTTTCAGTCTGAGGGCTCCAACAGTGATATGTATCTCAGCCCTGTTGCCATGTTTCGGGACCCCTTCCGCAGAGATCCCAACAAGCTGGTGTTCTGTGAAGTTTTCAAGTACAACCGGAAGCCCGCAGAGACCAATTTAAGGCACTCGTGTAAACGGATAATGGACATGGTGAGCAACCAGCACCCCTGGTTTGGAATGGAACAGGAGTATACTCTGATGGGAACAGATGGGCACCCTTTTGGTTGGCCTTCCAATGGCTTTCCTGGGCCCCAAGGTCCGTATTACTGTGGTGTGGGCGCAGACAAAGCCTATGGCAGGGACATCGTGGAGGCTCACTACCGCGCCTGCTTGTATGCTGGGGTCAAGATTACAGGAACAAATGCTGAGGTCATGCCTGCCCAGTGGGAATTCCAAATAGGACCCTGTGAAGGAATCCGCATGGGAGATCATCTCTGGGTGGCCCGTTTCATCTTGCATCGAGTATGTGAAGACTTTGGGGTAATAGCAACCTTTGACCCCAAGCCCATTCCTGGGAACTGGAATGGTGCAGGCTGCCATACCAACTTTAGCACCAAGGCCATGCGGGAGGAGAATGGTCTGAAGCACATCGAGGAGGCCATCGAGAAACTAAGCAAGCGGCACCGCTACCACATTCGAGCCTACGATCCCAAGGGGGGCCTGGACAATGCCCGTCGGCTGACTGGGTTCCACGAAACGTCCAACATCAACGACTTTTCTGCTGGTGTCGCCAATCGCAGTGCCAGCATCCGCATTCCCCGGACTGTCGGCCAGGAGAAGAAAGGTTACTTTGAAGACCGGCGCCCCTCTGCCAATTGTGACCCCTTTGCAGTGACAGAAGCCATCGTCCGCACATGCCTTCTCAATGAGACTGGCGACGAGCCCTTCCAATACAAAAACTAATTAGACTTTGAGTGATCTTGAGCCTTTCCTAGTTCATCCCACCCCGCCCCAGAGAAACACGGAAGGAGACAATACCGGAAGGAACCCGCGCTATGACGGCAATAAAAAGACAGAATAAAACGCACGGGTGTTGGGTCGTTTGTTCATAAACGCGGGGTTCGGTCCCAGGGCTGGCACTCTGTCGATACCCCACCGAGACCCCATTGGGGCCAATACGCCCGCGTTTCTTCCTTTTCCCCACCCCACCCCCCAAGTTCGGGTGAAGGCCCAGGGCTCGCAGCCAACGTCGGGGCGGCAGGCCCTGCCATTACCGTCGATCTCTAGCTAGGATCTTAAGCTGCAG[CMVpromoter]GTTGACATTGATTATTGACTAGTTATTAATAGTAATCAATTACGGGGTCATTAGTTCATAGCCCATATATGGAGTTCCGCGTTACATAACTTACGGTAAATGGCCCGCCTGGCTGACCGCCCAACGACCCCCGCCCATTGACGTCAATAATGACGTATGTTCCCATAGTAACGCCAATAGGGACTTTCCATTGACGTCAATGGGTGGAGTATTTACGGTAAACTGCCCACTTGGCAGTACATCAAGTGTATCATATGCCAAGTACGCCCCCTATTGACGTCAATGACGGTAAATGGCCCGCCTGGCATTATGCCCAGTACATGACCTTATGGGACTTTCCTACTTGGCAGTACATCTACGTATTAGTCATCGCTATTACCATGGTGATGCGGTTTTGGCAGTACATCAATGGGCGTGGATAGCGGTTTGACTCACGGGGATTTCCAAGTCTCCACCCCATTGACGTCAATGGGAGTTTGTTTTGGCACCAAAATCAACGGGACTTTCCAAAATGTCGTAACAACTCCGCCCCATTGACGCAAATGGGCGGTAGGCGTGTACGGTGGGAGGTCTATATAAGCAGAGCTCGTTTAGTGAACCGTCAGATCGCCTGGAGACGCCATCCACGCTGTTTTGACCTCCATAGAAGACACCGGGACCGATCCAGCCTCCGGACTCTA  AAGTTGGTCGTGAGGCACTGGGCAGGTAAGTATCAAGGTTACAAGACAGGTTTAAGGAGACCAATAGAAACTGGGCTTGTCGAGACAGAGAAGACTCTTGCGTTTCTGATAGGCACCTATTGGTCTTACTGACATCCACTTTGCCTTTCTCTCCACAGGTGTCCACTCCGAGCTCCTCGAGGGCTAGCTAAGCTTGCCACC[5B3light chain variable domain]ATGGGCTGGTCTTGCATAATCCTCTTTCTGGTAGCAACTGCAACCGGGGTACACAGTGATATACAAATGACCCAAAGTCCCTCTTCACTCTCTGCCAGTGTAGGAGATAGAGTTACTATCACCTGCCTGGCTTCTCAGACTATCGGTACTTGGCTCGCCTGGTACCAACAAAAACCCGGAAAGGCACCAAAGCTGCTTATCTATGCTGCTACATCCTTGGCTGATGGGGTCCCTTCTAGGTTTTCCGGCTCAGGAAGCGGTACCGATTTCACTTTGACCATTAGTTCTTTGCAACCTGAGGACTTTGCTACCTACTATTGTCAACAGTTCTATTCTACACCATTCACCTTTGGTGGCGGGACTAAGCTGGAGATAAAACGG[Bat light chain constant region] AACGTAGCTAAGCCTTCTACTTTCATATTTCCACCTAGTCAACAGCAACTCCAAACAGGAAAAGCCAGCGTCGTTTGTCTTCTCAACGGGTTCTACCCACGCGAAATAAATGTGAAGTGGAAGGTCGACGGAGTAGTCCAGACTAACGGCATACAGAACTCCGCTACCGAGCAAAATTCAAAGGACAACACCTACAGCTTGTCTTCAATTCTCACATTGAGTTCATCCGAATACAAATCCCACAATGTGTATGCATGTGAGATTACCCATCAAAGTTTGAACAGTGCCTTCGTGAAGTCCTTTAACCGGGAATCATGCTAA CCCCGGGGGATCCACGCGTCACCGGTTTCTAGACTCGGCGGCCGCGATTCGAACCCGCTGATCAGCCTCGA[BGH pA] *CTGTGCCTTCTAGTTGCCAGCCATCTGTTGTTT GCCCCTCCCCCGTGCCTTCCTTGACCCTGGAAGGTGCCAC TCCCACTGTCCTTTCCTAATAAAATGAGGAAATTGCATCGCATTGTCTGAGTAGGTGTCATTCTATTCTGGGGGGTGGGGTGGGGCAGGACAGCAAGGGGGAGGATTGGGAAGACAATAGCAGGCATGCTGGGGATGCGGTGGGCTCTATGG* AGATCTGTCGACCTCTAGCTAGAGCTTGGCGTAATCATGGTCATAGCTGTTTCCTGTGTGAAATTGTTATCCGCTCACAATTCCACACAACATACGAGCCGGAAGCATAAAGTGTAAAGCCTGGGGTGCCTAATGAGTGAGCTAACTCACATTAATTGCGTTGCGCTCACTGCCCGCTTTCCAGTCGGGAAACCTGTCGTGCCAGCTGCATTAATGAATCGGCCAACGCGCGGGGAGAGGCGGTTTGCGTATTGGGCGCTCTTCCGCTTCCTCGCTCACTGACTCGCTGCGCTCGGTCGTTCGGCTGCGGCGAGCGGTATCAGCTCACTCAAAGGCGGTAATACGGTTATCCACAGAATCAGGGGATAACGCAGGAAAGAACATGTGAGCAAAAGGCCAGCAAAAGGCCAGGAACCGTAAAAAGGCCGCGTTGCTGGCGTTTTTCCATAGGCTCCGCCCCCCTGACGAGCATCACAAAAATCGACGCTCAAGTCAGAGGTGGCGAAACCCGACAGGACTATAAAGATACCAGGCGTTTCCCCCTGGAAGCTCCCTCGTGCGCTCTCCTGTTCCGACCCTGCCGCTTACCGGATACCTGTCCGCCTTTCTCCCTTCGGGAAGCGTGGCGCTTTCTCATAGCTCACGCTGTAGGTATCTCAGTTCGGTGTAGGTCGTTCGCTCCAAGCTGGGCTGTGTGCACGAACCCCCCGTTCAGCCCGACCGCTGCGCCTTATCCGGTAACTATCGTCTTGAGTCCAACCCGGTAAGACACGACTTATCGCCACTGGCAGCAGCCACTGGTAACAGGATTAGCAGAGCGAGGTATGTAGGCGGTGCTACAGAGTTCTTGAAGTGGTGGCCTAACTACGGCTACACTAGAAGAACAGTATTTGGTATCTGCGCTCTGCTGAAGCCAGTTACCTTCGGAAAAAGAGTTGGTAGCTCTTGATCCGGCAAACAAACCACCGCTGGTAGCGGTTTTTTTGTTTGCAAGCAGCAGATTACGCGCAGAAAAAAAGGATCTCAAGAAGATCCTTTGATCTTTTCTACGGGGTCTGACGCTCAGTGGAACGAAAACTCACGTTAAGGGATTTTGGTCATGAGATTATCAAAAAGGATCTTCACCTAGATCCTTTTAAATTAAAAATGAAGTTTTAAATCAATCTAAAGTATATATGAGTAAACTTGGTCTGACAG [Amp (R)] TTACCAATGCTTAATCAGTGAGGCACCTATCTCAGCGATCTGTCTATTTCGTTCATCCATAGTTGCCTGACTCCCCGTCGTGTAGATAACTACGATACGGGAGGGCTTACCATCTGGCCCCAGTGCTGCAATGATACCGCGAGACCCACGCTCACCGGCTCCAGATTTATCAGCAATAAACCAGCCAGCCGGAAGGGCCGAGCGCAGAAGTGGTCCTGCAACTTTATCCGCCTCCATCCAGTCTATTAATTGTTGCCGGGAAGCTAGAGTAAGTAGTTCGCCAGTTAATAGTTTGCGCAACGTTGTTGCCATTGCTACAGGCATCGTGGTGTCACGCTCGTCGTTTGGTATGGCTTCATTCAGCTCCGGTTCCCAACGATCAAGGCGAGTTACATGATCCCCCATGTTGTGCAAAAAAGCGGTTAGCTCCTTCGGTCCTCCGATCGTTGTCAGAAGTAAGTTGGCCGCAGTGTTATCACTCATGGTTATGGCAGCACTGCATAATTCTCTTACTGTCATGCCATCCGTAAGATGCTTTTCTGTGACTGGTGAGTACTCAACCAAGTCATTCTGAGAATAGTGTATGCGGCGACCGAGTTGCTCTTGCCCGGCGTCAATACGGGATAATACCGCGCCACATAGCAGAACTTTAAAAGTGCTCATCATTGGAAAACGTTCTTCGGGGCGAAAACTCTCAAGGATCTTACCGCTGTTGAGATCCAGTTCGATGTAACCCACTCGTGCACCCAACTGATCTTCAGCATCTTTTACTTTCACCAGCGTTTCTGGGTGAGCAAAAACAGGAAGGCAAAATGCCGCAAAAAAGGGAATAAGGGCGACACGGAAATGTTGAATACTCAT [bla promoter] ACTCTTCCTTTTTCAATATTATTGAAGCATTTATCAGGGTTATTGTCTCATGAGCGGATACATATTTGAATGTATTTAGAAAAATAAACAAATAGGGGT TCCGCGCACATTTCCCCGAAAAGTGCCACCTGACGTC |
| --- | --- |
| pNBF Bat 5B3 heavy chain IgG1 with Bsp or Hsp | GACGTTTAAACAATTCCTTAGGCTACCGGGTAGGGGAGGCGCTTTTCCCAAGGCAGTCTGGAGCATGCGCTTTAGCAGCCCCGCTGGGCACTTGGCGCTACACAAGTGGCCTCTGGCCTCGCACACATTCCACATCCACCGGCCGGTAGGCGCCAACCGGCTCCGTTCTTTGGTGGCCCCTTCGCGCCACCTTCTACTCCTCCCCTAGTCAGGAAGTTCCCCCCCGCCCCGCAGCTCGCGTCGTGCAGGACGTGACAAATGGAAGTAGCACGTCTCACTAGTCTCGTGCAGATGGACAGCACCGCTGAGCAATGGAAGCGGGTAGGCCTTTGGGGCAGCGGCCAATAGCAGCTTTGCTCCTTCGCTTTCTGGGCTCAGAGGCTGGGAAGGGGTGGGTCCGGGGGCGGGCTCAGGGGCGGGCTCAGGGGCGGGGCGGGCGCCCGAAGGTCCTCCGGAGGCCCGGCATTCTGCACGCTTCAAAAGCGCACGTCTGCCGCGCTGTTCTCCTCTTCCTCATCTCCGGGCCTTTCGACCAGCTTACC[Puromycin] ATGACCGAGTACAAGCCCACGGTGCGCCTCGCCACCCGCGACGACGTCCCCAGGGCCGTACGCACCCTCGCCGCCGCGTTCGCCGACTACCCCGCCACGCGCCACACCGTCGATCCGGACCGCCACATCGAGCGGGTCACCGAGCTGCAAGAACTCTTCCTCACGCGCGTCGGGCTCGACATCGGCAAGGTGTGGGTCGCGGACGACGGCGCCGCGGTGGCGGTCTGGACCACGCCGGAGAGCGTCGAAGCGGGGGCGGTGTTCGCCGAGATCGGCCCGCGCATGGCCGAGTTGAGCGGTTCCCGGCTGGCCGCGCAGCAACAGATGGAAGGCCTCCTGGCGCCGCACCGGCCCAAGGAGCCCGCGTGGTTCCTGGCCACCGTCGGCGTCTCGCCCGACCACCAGGGCAAGGGTCTGGGCAGCGCCGTCGTGCTCCCCGGAGTGGAGGCGGCCGAGCGCGCCGGGGTGCCCGCCTTCCTGGAGACCTCCGCGCCCCGCAACCTCCCCTTCTACGAGCGGCTCGGCTTCACCGTCACCGCCGACGTCGAGGTGCCCGAAGGACCGCGCACCTGGTGCATGACCCGCAAGCCCGGTGCCTGACGCCCGCCCCACGACCCGCAGCGCCCGACCGAAAGGAGCGCACGACCCCATGCATCGTAGAATTCCGGACTTGACTCATGCTTGTTTCACTTTCACATGGAATTTCCCAGTTATGAAATTAATAAAAATCAATGGTTTCCACATCTCTCAGTGCCTCTATCTGGAGGCCAGGTAGGGCTGGCCTTGGGGGAGGGGGAGGCCAGAATGACTCCAAGAGCTACAGGAAGGCAGGTCAGAGACCCCACTGGACAAACAGTGGCTGGACTCTGCACCATAACACACAATCAACAGGGGAGTGAGCTGGAGGGGGATCGTTTAAACCTTAAGATATCCAG[SV40 Promoter] CATCTCAATTAGTCAGCAACCATAGTCCCGCCCCTAACTCCGCCCATCCCGCCCCTAACTCCGCCCAGTTCCGCCCATTCTCCGCCCCATGGCTGACTAATTTTTTTTATTTATGCAGAGGCCGAGGCCGCCTCTGCCTCTGAGCTAGTCGCGATTGATCAGCGCCACCTTGGGGCCACCGCTCAGAGCACCTTCCACC [GS gene] ATGGCCACCTCAGCAAGTTCCCACTTGAACAAAAACATCAAGCAAATGTACTTGTGCCTGCCCCAGGGTGAGAAAGTCCAAGCCATGTATATCTGGGTTGATGGTACTGGAGAAGGACTGCGCTGCAAAACCCGCACCCTGGACTGTGAGCCCAAGTGTGTAGAAGAGTTACCTGAGTGGAATTTTGATGGCTCTAGTACCTTTCAGTCTGAGGGCTCCAACAGTGATATGTATCTCAGCCCTGTTGCCATGTTTCGGGACCCCTTCCGCAGAGATCCCAACAAGCTGGTGTTCTGTGAAGTTTTCAAGTACAACCGGAAGCCCGCAGAGACCAATTTAAGGCACTCGTGTAAACGGATAATGGACATGGTGAGCAACCAGCACCCCTGGTTTGGAATGGAACAGGAGTATACTCTGATGGGAACAGATGGGCACCCTTTTGGTTGGCCTTCCAATGGCTTTCCTGGGCCCCAAGGTCCGTATTACTGTGGTGTGGGCGCAGACAAAGCCTATGGCAGGGACATCGTGGAGGCTCACTACCGCGCCTGCTTGTATGCTGGGGTCAAGATTACAGGAACAAATGCTGAGGTCATGCCTGCCCAGTGGGAATTCCAAATAGGACCCTGTGAAGGAATCCGCATGGGAGATCATCTCTGGGTGGCCCGTTTCATCTTGCATCGAGTATGTGAAGACTTTGGGGTAATAGCAACCTTTGACCCCAAGCCCATTCCTGGGAACTGGAATGGTGCAGGCTGCCATACCAACTTTAGCACCAAGGCCATGCGGGAGGAGAATGGTCTGAAGCACATCGAGGAGGCCATCGAGAAACTAAGCAAGCGGCACCGCTACCACATTCGAGCCTACGATCCCAAGGGGGGCCTGGACAATGCCCGTCGGCTGACTGGGTTCCACGAAACGTCCAACATCAACGACTTTTCTGCTGGTGTCGCCAATCGCAGTGCCAGCATCCGCATTCCCCGGACTGTCGGCCAGGAGAAGAAAGGTTACTTTGAAGACCGGCGCCCCTCTGCCAATTGTGACCCCTTTGCAGTGACAGAAGCCATCGTCCGCACATGCCTTCTCAATGAGACTGGCGACGAGCCCTTCCAATACAAAAACTAATTAGACTTTGAGTGATCTTGAGCCTTTCCTAGTTCATCCCACCCCGCCCCAGAGAAACACGGAAGGAGACAATACCGGAAGGAACCCGCGCTATGACGGCAATAAAAAGACAGAATAAAACGCACGGGTGTTGGGTCGTTTGTTCATAAACGCGGGGTTCGGTCCCAGGGCTGGCACTCTGTCGATACCCCACCGAGACCCCATTGGGGCCAATACGCCCGCGTTTCTTCCTTTTCCCCACCCCACCCCCCAAGTTCGGGTGAAGGCCCAGGGCTCGCAGCCAACGTCGGGGCGGCAGGCCCTGCCATTACCGTCGATCTCTAGCTAGGATCTTAAGCTGCAG[CMVpromoter]GTTGACATTGATTATTGACTAGTTATTAATAGTAATCAATTACGGGGTCATTAGTTCATAGCCCATATATGGAGTTCCGCGTTACATAACTTACGGTAAATGGCCCGCCTGGCTGACCGCCCAACGACCCCCGCCCATTGACGTCAATAATGACGTATGTTCCCATAGTAACGCCAATAGGGACTTTCCATTGACGTCAATGGGTGGAGTATTTACGGTAAACTGCCCACTTGGCAGTACATCAAGTGTATCATATGCCAAGTACGCCCCCTATTGACGTCAATGACGGTAAATGGCCCGCCTGGCATTATGCCCAGTACATGACCTTATGGGACTTTCCTACTTGGCAGTACATCTACGTATTAGTCATCGCTATTACCATGGTGATGCGGTTTTGGCAGTACATCAATGGGCGTGGATAGCGGTTTGACTCACGGGGATTTCCAAGTCTCCACCCCATTGACGTCAATGGGAGTTTGTTTTGGCACCAAAATCAACGGGACTTTCCAAAATGTCGTAACAACTCCGCCCCATTGACGCAAATGGGCGGTAGGCGTGTACGGTGGGAGGTCTATATAAGCAGAGCTCGTTTAGTGAACCGTCAGATCGCCTGGAGACGCCATCCACGCTGTTTTGACCTCCATAGAAGACACCGGGACCGATCCAGCCTCCGGACTCTA  AAGTTGGTCGTGAGGCACTGGGCAGGTAAGTATCAAGGTTACAAGACAGGTTTAAGGAGACCAATAGAAACTGGGCTTGTCGAGACAGAGAAGACTCTTGCGTTTCTGATAGGCACCTATTGGTCTTACTGACATCCACTTTGCCTTTCTCTCCACAGGTGTCCACTCCGAGCTCCTCGAGGGCTAGCTAAGCTTGCCACC  **[Bsp] ATGGACAGGAGTTGGCGGGTTCTGCTGTTGGTGGCTGTTGCCACAGGGGTACATGCCGAG or**  **[Hsp] ATGGGATGGAGCTGTATCATCCTCTTCTTGGTAGCAACAGCTACAGGTGTCCACTCTGAG** GTTCAACTTGTGGAATCTGGGGGCGGCCTCGTCAAGCCAGGAGGATCCCTGCGGCTTTCCTGTGCCGCAAGTGGCTTCACATTTTCTTCCTATGACATGAGCTGGGTTCGCCAAGCCCCTGGCAAAGGATTGGAATGGGTGGCAATGATCTCCTCAGGGGGAAGTTACTCCTACTACCCCGACAGTGTAAAAGGAAGATTCACAATATCACGAGACAACTCCAAGAACACTCTTTACTTGCAGATGAACTCACTGCGTGCTGAAGATACTGCTGTTTATTATTGCGCTCGACAAGGAGACTATGCTTGGTTTGCTTACTGGGGACAGGGGACTCTCGTCACCGTTAGCAGC  [Bat heavy chain chain constant region] GCTACCACAAAGGCACCCTCAGTCTTTCCACTCAGTCCTCCAAAAGAGGCTTCATCCGAAAGTACCATTTCACTTGCTTGTCTCGTTTCCGGATTCTTCCCTGAGCCTGTCACCGTGTCATGGAATTCAGGCGCACTCAGTTCCGGCGTATACACATTCCCAAGTTCCTTGCATAGCTCCGGACTCTATTCCCAATCAAGTATGGTAACAGTACCTAAAAGCTCCGCCTCCGGACAGACATATACATGCAATGTGGCACATCCTGCTTCATCAACTAAAGTAGACAAGATAGTTCCAGTAAAATACACATGTGATAATGGGGGCAACCCATGTCCTGCTCCAGACCTTCTGGGTGGCCCTTCAGTCTTTATATTTCCCCCAAAGGCCAGAGACACTTTGATGATATCCCGTACACCAGAAGTCACTTGTGTAGTGGTCGACGTATCCCAGGACGATACAAACATCAAGTTTACATGGTATGTAGATGGTAAGCAATCTAATATGGGTAAGACCAAGGAGCCTGAGGAACAGTTTAATTCCACTTATCGAATTGTGTCTGTCCTCCCAATTTTGCACCAAGACTGGCTTAATGGGAAGCAATTTAAATGTAAGGTCAATTCAAAAGCCCTGCCAGCCCCAATAGAACGTACTATTACCAAGGCAAAGGGACCTATTCGTGAACCACATGTCTATGTTCTCAACCCTCATCCCGACGAGCTCGCCAAAGACGTAGTTAGTGTCACTTGCTTGGTGAAAGGGTTTTACCCCCCTGACATAAACGTGGAGTGGGAATCCAACGAACAACCCGAGCCAGAGGCCAAATACCAGACTACACCAGCACAGCTTGATGCCGACGGATCTTTCTTTCTCTACAGTAAACTTACCGTGGAAAAAAGCAGATGGCAACGAGGCCAAGATTTCACTTGTACTGTCATGCATGAGGCCCTGCACAATCACTATACCCAGAAATCAAGTTCTCATACAAGCGGCTAA  [BGH pA] *ACTCGGCGGCCGCGATTCGAACCCGCTGATCAGCCTCGA*  *CTGTGCCTTCTAGTTGCCAGCCATCTGTTGTTTGCCCCTCCCCCGTGCCTTCCTTGACCCTGGAAGGTGCCACTCCCACTGTCCTTTCCTAATAAAATGAGGAAATTGCATCGCATTGTCTGAGTAGGTGTCATTCTATTCTGGGGGGTGGGGTGGGGCAGGACAGCAAGGGGGAGGATTGGGAAGACAATAGCAGGCATGCTGGGGATGCGGTGGGCTCTATGG*  AGATCTGTCGACCTCTAGCTAGAGCTTGGCGTAATCATGGTCATAGCTGTTTCCTGTGTGAAATTGTTATCCGCTCACAATTCCACACAACATACGAGCCGGAAGCATAAAGTGTAAAGCCTGGGGTGCCTAATGAGTGAGCTAACTCACATTAATTGCGTTGCGCTCACTGCCCGCTTTCCAGTCGGGAAACCTGTCGTGCCAGCTGCATTAATGAATCGGCCAACGCGCGGGGAGAGGCGGTTTGCGTATTGGGCGCTCTTCCGCTTCCTCGCTCACTGACTCGCTGCGCTCGGTCGTTCGGCTGCGGCGAGCGGTATCAGCTCACTCAAAGGCGGTAATACGGTTATCCACAGAATCAGGGGATAACGCAGGAAAGAACATGTGAGCAAAAGGCCAGCAAAAGGCCAGGAACCGTAAAAAGGCCGCGTTGCTGGCGTTTTTCCATAGGCTCCGCCCCCCTGACGAGCATCACAAAAATCGACGCTCAAGTCAGAGGTGGCGAAACCCGACAGGACTATAAAGATACCAGGCGTTTCCCCCTGGAAGCTCCCTCGTGCGCTCTCCTGTTCCGACCCTGCCGCTTACCGGATACCTGTCCGCCTTTCTCCCTTCGGGAAGCGTGGCGCTTTCTCATAGCTCACGCTGTAGGTATCTCAGTTCGGTGTAGGTCGTTCGCTCCAAGCTGGGCTGTGTGCACGAACCCCCCGTTCAGCCCGACCGCTGCGCCTTATCCGGTAACTATCGTCTTGAGTCCAACCCGGTAAGACACGACTTATCGCCACTGGCAGCAGCCACTGGTAACAGGATTAGCAGAGCGAGGTATGTAGGCGGTGCTACAGAGTTCTTGAAGTGGTGGCCTAACTACGGCTACACTAGAAGAACAGTATTTGGTATCTGCGCTCTGCTGAAGCCAGTTACCTTCGGAAAAAGAGTTGGTAGCTCTTGATCCGGCAAACAAACCACCGCTGGTAGCGGTTTTTTTGTTTGCAAGCAGCAGATTACGCGCAGAAAAAAAGGATCTCAAGAAGATCCTTTGATCTTTTCTACGGGGTCTGACGCTCAGTGGAACGAAAACTCACGTTAAGGGATTTTGGTCATGAGATTATCAAAAAGGATCTTCACCTAGATCCTTTTAAATTAAAAATGAAGTTTTAAATCAATCTAAAGTATATATGAGTAAACTTGGTCTGACAG[Amp (R)] TTACCAATGCTTAATCAGTGAGGCACCTATCTCAGCGATCTGTCTATTTCGTTCATCCATAGTTGCCTGACTCCCCGTCGTGTAGATAACTACGATACGGGAGGGCTTACCATCTGGCCCCAGTGCTGCAATGATACCGCGAGACCCACGCTCACCGGCTCCAGATTTATCAGCAATAAACCAGCCAGCCGGAAGGGCCGAGCGCAGAAGTGGTCCTGCAACTTTATCCGCCTCCATCCAGTCTATTAATTGTTGCCGGGAAGCTAGAGTAAGTAGTTCGCCAGTTAATAGTTTGCGCAACGTTGTTGCCATTGCTACAGGCATCGTGGTGTCACGCTCGTCGTTTGGTATGGCTTCATTCAGCTCCGGTTCCCAACGATCAAGGCGAGTTACATGATCCCCCATGTTGTGCAAAAAAGCGGTTAGCTCCTTCGGTCCTCCGATCGTTGTCAGAAGTAAGTTGGCCGCAGTGTTATCACTCATGGTTATGGCAGCACTGCATAATTCTCTTACTGTCATGCCATCCGTAAGATGCTTTTCTGTGACTGGTGAGTACTCAACCAAGTCATTCTGAGAATAGTGTATGCGGCGACCGAGTTGCTCTTGCCCGGCGTCAATACGGGATAATACCGCGCCACATAGCAGAACTTTAAAAGTGCTCATCATTGGAAAACGTTCTTCGGGGCGAAAACTCTCAAGGATCTTACCGCTGTTGAGATCCAGTTCGATGTAACCCACTCGTGCACCCAACTGATCTTCAGCATCTTTTACTTTCACCAGCGTTTCTGGGTGAGCAAAAACAGGAAGGCAAAATGCCGCAAAAAAGGGAATAAGGGCGACACGGAAATGTTGAATACTCAT [bla promoter] ACTCTTCCTTTTTCAATATTATTGAAGCATTTATCAGGGTTATTGTCTCATGAGCGGATACATATTTGAATGTATTTAGAAAAATAAACAAATAGGGGT TCCGCGCACATTTCCCCGAAAAGTGCCACCTGACGTC |
| Plasmid maps of the pNBF expression vector encoding the (A) 5B3 light-chain variable domain and P. alecto light chain constant region (B) 5B3 heavy-chain variable domain and P. alecto IgG1 constant region. | **A**  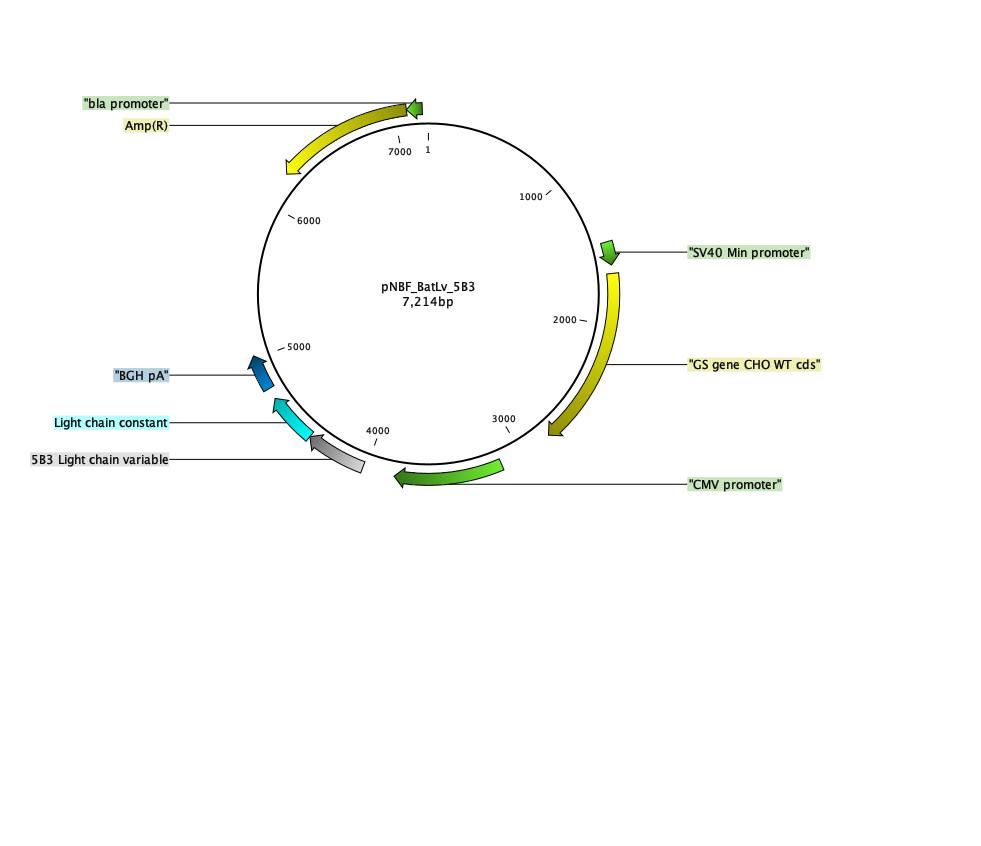  **B**  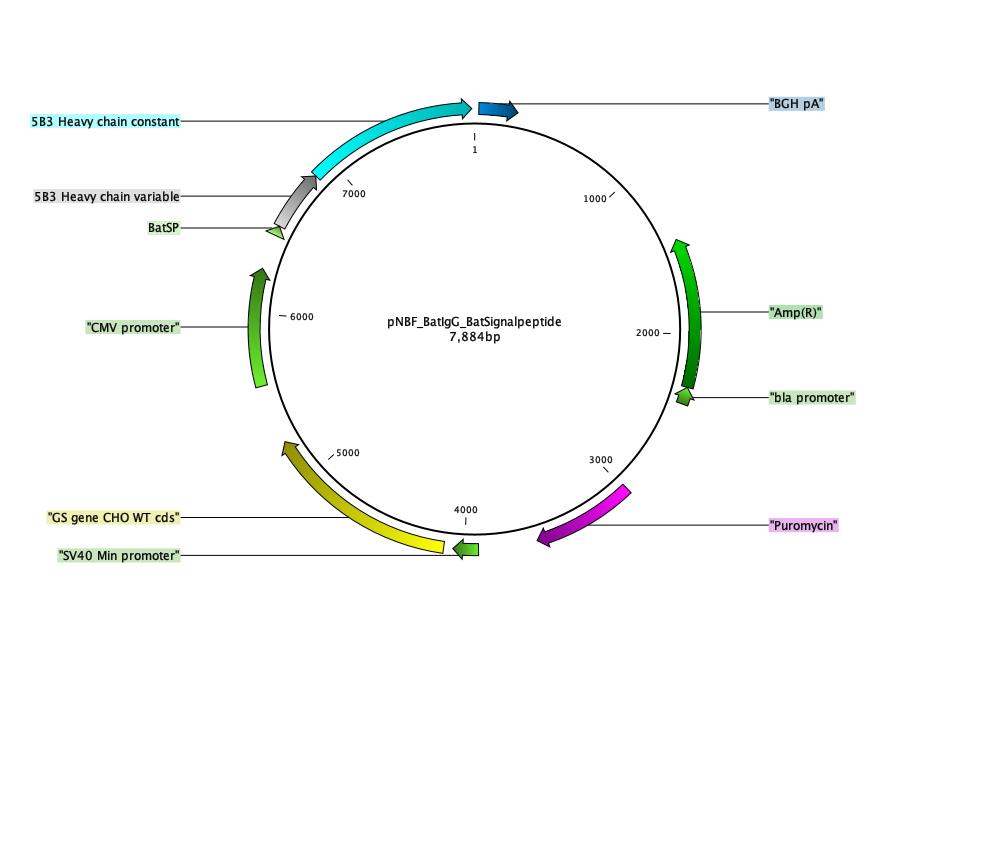 |
